# Robotic dog for navigation of a rehabilitation wheelchair robot in a highly constrained environment

**DOI:** 10.1371/journal.pone.0310024

**Published:** 2024-09-20

**Authors:** Bibhya Sharma

**Affiliations:** School of Information Technology, Engineering, Mathematics & Physics, The University of the South Pacific, Suva, Fiji; Xi’an Jiaotong University, CHINA

## Abstract

Adaptation to technological advancements and intelligent digital tools can enable healthcare providers to overcome the challenges of their patient-oriented care systems and processes. One such intelligent tool is automated assistive robots, which can improve patient care and safety in the health sector. This paper presents an invariant set of continuous nonlinear control laws for an assistive robot and a rehabilitation wheelchair robot modeled as a new autonomous robotic dog and rehabilitation wheelchair system for navigating a highly constrained environment. The control laws are derived from the Lyapunov-based control scheme classified under the umbrella of artificial potential field (APF) methods, and inherently proved stability of the new heterogeneous system. The robotic dog guides the wheelchair robot during the navigation process in a cluttered environment where the avoidances are from the robotic dog and the integrated dynamic protective polygon. The wheelchair traverses the obstacle-free path traced by the dynamic polygon. The leash is flexible, and its length is bounded, which invariably provides the protective polygon to change its intrinsic dimension. Thus, the dual-robot system has increased mobility for obstacle avoidance and passing through narrow passageways. The solution proffered herein is only feasible in a highly constrained and isolated human environment where nothing else appears to be moving in the direction of the robotic dog and wheelchair. The computer simulations and associated convergence graphs present the efficacy of the unique control laws for the new heterogeneous robotic system. Adoption of such control laws and their suitable variants can make a big impact in the healthcare industry.

## 1 Introduction

This Modern-day science and ICT (Information and Communications Technology) have made possible the impossible with the introduction of innovative devices, tools and technologies for the betterment of human survival and livelihood. These advances have also brought normalcy and relief to those who are physically and mentally challenged, or have some form of health defects. When available and accessible, ICT can logically and critically permit persons with disabilities (PWD) to understand practical opportunities to participate equally in all aspects of society and development [1]. Inclusion of PWD is possible by promoting research and development on innovative ICTs, which could be made available at an affordable value [2]. These ICT-driven devices or assistive technologies can help PWD have greater access to education, health care facilities and governance, reducing dependency on others.

One of the assistive technologies that has improved the living standards for persons with motor disabilities is a wheelchair, as nearly 1% of the world population uses it [3]. The earliest wheelchair was operated almost entirely by the individual, usually with weak motor skills, which added much pressure to them with every push or turn. Hence, researchers focused on developing smart wheelchairs. The button-based wheelchair eases the difficulty as disabled people can operate by pressing buttons on its keypad commanding the wheelchair to its destination. The voice-based wheelchair can also assist the physically challenged [4]. With limited movement, a person’s voice is ideal for operating the wheelchair. The person voices the command to an android mobile, which then transmits it to the microcontroller in the form of text using Bluetooth [3, 5–7]. However, for those individuals who may not speak, the gesture-based wheelchair is ideal for them. For instance, people suffering from paraplegia can give directions with their hands. Thus, the hand gesture-controlled wheelchair is most suitable for them for relocation. The hand gesture-based wheelchair’s proficiency lies in its sensor that reads the hand gesture indicated by the user and, consequently, moves in that direction. For a frail child, the joystick-based wheelchair was assembled. The motor controls the joystick, which the child commands to maneuver the wheelchair [3, 5]. Nonetheless, the eyeball-sensed wheelchair [3, 8] surpasses all others as it supports those with a severe form of paralysis where only a few muscular movements are possible including that of the eyeballs which the person can still control.

The control of wheelchairs can also be maintained using EEG signals. Deliberate blinking sends concentrated EEG signals from the occipital section of the brain known as scalp EEG to command the movement of the wheelchair. When a person blinks, the collection signal will illustrate a small peak whereas intentional blinking illustrates a higher peak. An appropriate threshold is determined between normal and intentional blinking to determine how the wheelchair will move [9]. To add on, as reported in [10] assistive tongue drive systems (TDS) could be used to interact with an environment through android devices and could easily be integrated to control a wheelchair system.

**Fig 1:**
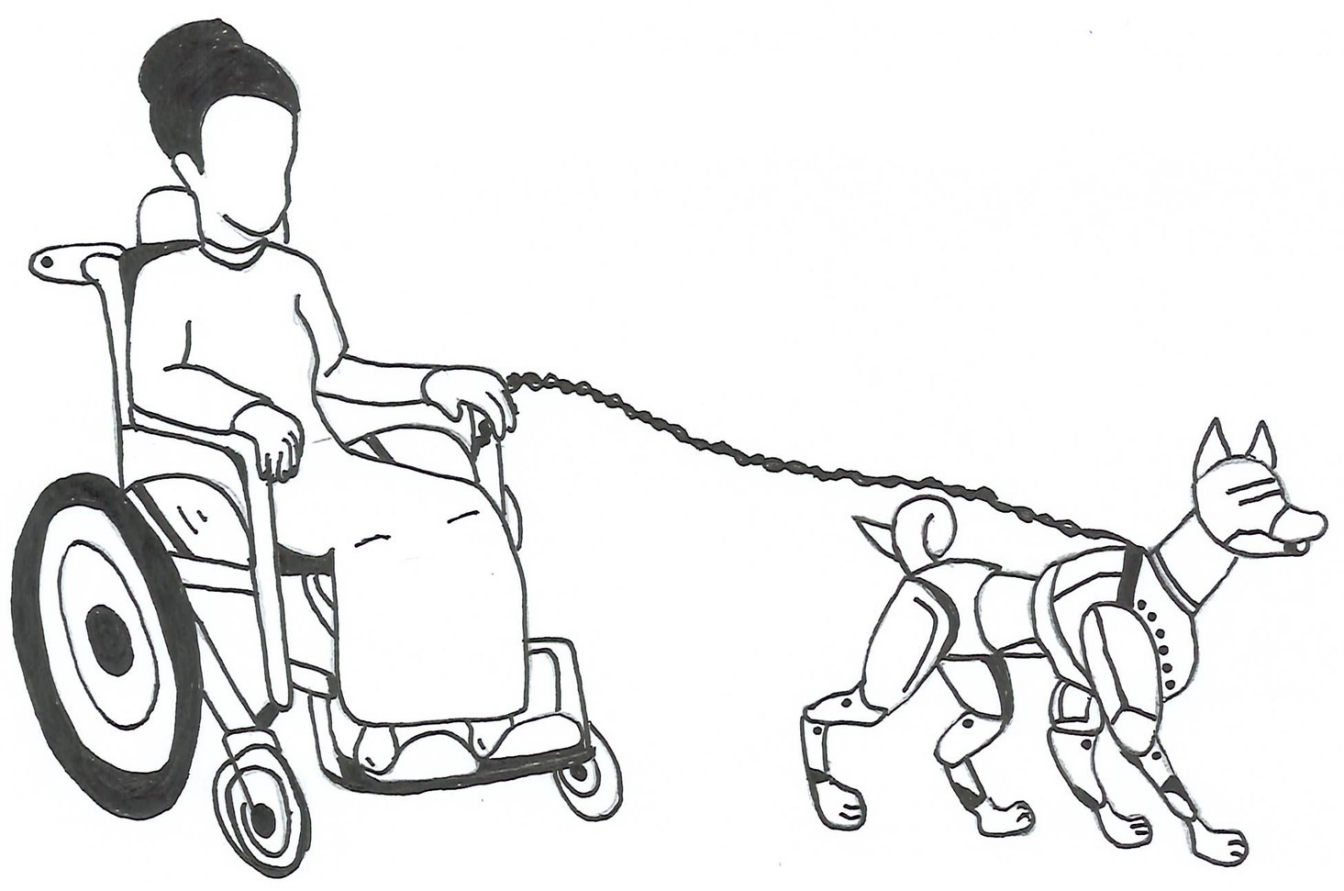
Schematic diagram of a guide dog assisting a PWD on a wheelchair.

All-in-all, wheelchairs in the literature mainly need human interaction for them to be able to move to a destination whilst avoiding obstacles. However, a wheelchair that an assisting robot can guide in a highly constrained environment without human interaction has not been given due attention. Motivated by the gaps in the literature, in this paper acceleration-based controllers are derived using an artificial potential fields (APFs) approach, known as Lyapunov based control Scheme (LbCS) for a new autonomous robotic dog wheelchair system where a virtual flexible but bounded leash connects the two robots (see Fig 1). The stabilizing time-invariant nonlinear controllers will enable the autonomous system to navigate an environment cluttered with obstacles without any localised input or intervention from the user of the wheelchair. The communication is assumed to be preprogrammed for daily routines by the supervisor in consultation with the people with disabilities riding the wheelchair. Essentially, this system observes the snow plough analogy from [11], where a virtual point establishes a collision and obstacle avoidance-free path for multi-agents in formation with its protective region to do the job of a snow plough. The formation is unchanged since the path traced by the snow plough is obstacle-free, establishing a rigid global formation of the multi-agents. A similar strategy is proposed for this research; however, a dynamical protective polygonal is used instead of a snow plough for the protection. As a result, the robotic dog and the dynamical protective polygon together trace an obstacle-free region for the wheelchair to take a path in. The leash is flexible, and its length is bounded, which invariably provides the protective polygon to change its intrinsic dimension. Thus, the dual-robot system has increased mobility for obstacle avoidance and passing through narrow passageways. We note that the advantages of the LbCS are easier implementation and more convenient analytic representations of system singularities and inequalities. Finally, the Direct Method of Lyapunov is employed to establish the stability of the new system.

While the literature has a plethora of research on motion planning and control of various robotic systems, this paper introduces a novel concept of mobilizing a wheelchair system in a highly constrained environment with the guidance of a robotic dog, essentially a new variant leader-follower strategy. The author believes that it is the first time within the framework of LbCS that such a motion control problem of the dual robotic system is considered with nonlinear continuous acceleration-based control laws. Furthermore, the robotic dog integrated into a new dynamic polygonal protective region finds safer and unobstructed region for the wheelchair to establish its own path without any interaction or intervention from the user of the wheelchair. Another contribution is the unique mathematical treatment of the Minimum Distance Technique (MDT), which guarantees simpler and relatively easier computations of the nonlinear control laws.

The layout of the remainder of the paper is as follows: Section 2 presents a discussion on the different types of assistive robots in the health care system. Next, a brief description of the LbCS is provided in Section 3, and the system modeling of a point-based robotic dog and a wheelchair robot is given in Section 4. Subsequently, Section 5 provides the attractive and repulsive artificial potential functions for target attraction and obstacle avoidance. Next, in Section 6, the nonlinear control laws derived from a Lyapunov function are presented with the stability analysis of the new autonomous robotic dog and rehabilitation wheelchair system. Then, the simulation results are presented in Section 7, and the discussion and conclusion are in Section 8 and Section 9, respectively.

## 2 Assistive robots in health care

The sharp rise of the aging community and people with disabilities (PWD), lack of qualified nursing personnel, and the public’s varying expectations of public health care systems impend the quality of patient-centered care in health care amenities [12]. Other challenges include poor communication between the aging community or PWDs and the healthcare providers regarding their safe and directed transportation. However, intelligent adaptation to technological advancements could enable healthcare providers to solve issues concerning patient-centered care processes. One such technological advancement is assistive robots, which has recently improved patient-centered care in the health sector. Monitoring, supporting, transporting, assessing, and stimulating patients, the elderly, and persons with disabilities are the primary focus.

Assistive robots can be classified as interactive and noninteractive robots. Noninteractive assistive robots are commonly seen in surgical theaters, rehabilitation programs, and for the transportation of medicines. Surgeons directly control surgical robots, which lack artificial intelligence and are not automated. The rehabilitation robots are seen as assistive tools that aid people with mobility limitations and are generally of two types: (a) home-use systems (like smart wheelchairs, artificial limbs, exoskeleton, and assistive manipulators), which assist a person in their daily living, and (b) therapeutic systems (devices that helps the rehabilitation process, for instance, the restoration of hand and arm motion function after stroke and spinal cord injury) used in clinical facilities. Finally, there are medical delivery robots, which are automated drug dispensing devices that relieve nurse shortages, improve productivity, and reduce medication errors. Such automated robots play a huge role in emergencies and crises such as the COVID-19 pandemic.

In comparison, interactive robots, also known as Socially assistive robots (SAR), can be categorized into service robots and companion robots (animal-like and human-like) [13]. The service robots give essential comfort for independent living (such as eating, dressing, toiletry, and bathing), whereas companion robots give social companionship to individuals, enhancing the individual’s health and psychological well-being. An individual can also relate their emotions to companion robots. However, for successful development and deployment of SAR, the social context in which they will be deployed needs to be considered [14].

Over the last two decades, a dearth of attention has been given to service and companion robots, for example robotic guide dogs (RGD) [15–17]. In the work [15], an initiative was made to replace guide dogs, which are costly and time-consuming to train, with an RGD for a relatively short service life. A Bioloid robotic dog from Robotis was selected with three directional infrared sensors and distance, brightness, sound, and temperature detecting abilities. Finally, voice recognition was implemented and interfaced with the robot to guide a visually-impaired person. In 2020, a final-year student at Loughborough University introduced the handheld RGD called Theia for visually-impaired individuals [16]. Theia guides its user’s hand utilizing force feedback through a control moment gyroscope, which is as good as holding a guide dog’s brace. Through a voice command, a Theia user must state the destination, and then with minimal user input, Theia guides its user in a large indoor and outdoor environment. Furthermore, a quadrupedal robot called a Mini Cheetah was utilized to develop an RGD in [17] for the visually-impaired individual through a taut and slack leash, which provides the ability to switch the intrinsic dimension of the human-robot system. Furthermore, since the system is not rigid it significantly increases mobility in confined spaces.

This research considers a service and a companion robot called a robotic dog, leashed to a wheelchair system to design an automated robotic dog wheelchair robot system to consider safe transportation and uninterrupted companionship of visually-impaired individuals also of those who have mobility disabilities. In the current research, the leash is flexible but its length is bounded which invariably provides the integrated dynamic protective polygon of the system to change its intrinsic dimension. Thus, the dual-robot autonomous system has increased mobility for obstacle avoidance and passing through narrow passageways. Another advantage of the new system is that the wheelchair has its own path in the obstacle-free region traced by the dynamic polygon in a highly constrained environment.

## 3 Lyapunov-based control scheme

The Lyapunov-based control scheme (LbCS) proposed by Sharma in [18], an artificial potential field method categorized as a classical approach is utilized in this research. The primary concept of the scheme is developing a suitable total potential known as a Lyapunov function which operates as an energy function. It involves the construction of avoidance and attractive functions for repulsion from the obstacle(s) and attraction to target(s), respectively. A repulsive potential function is a ratio of the product of an auxiliary function and a positive *tuning parameter* to the obstacle avoidance function. The sum of all repulsive and attractive potential functions is called the Lyapunov function. The control laws derived from the total potential ensure that for all *t* ≥ 0, the Lyapunov function decreases and vanishes to zero as *t* → ∞. The main strength of LbCS is the easy and elegant design of controllers that are also continuous at all times *t* ≥ 0. Additionally, the specifications, control conditions, constraints of mechanical systems and inequalities can easily be incorporated into the LbCS through an ingenious construction of artificial obstacles. For a thorough understanding of the LbCS, the reader is referred to [18]. The nonlinear velocity or acceleration time-invariant controllers extracted from LbCS have been successfully applied in the literature to find stabilizing and feasible solutions to an array of problems [19–30].

**Fig 2:**
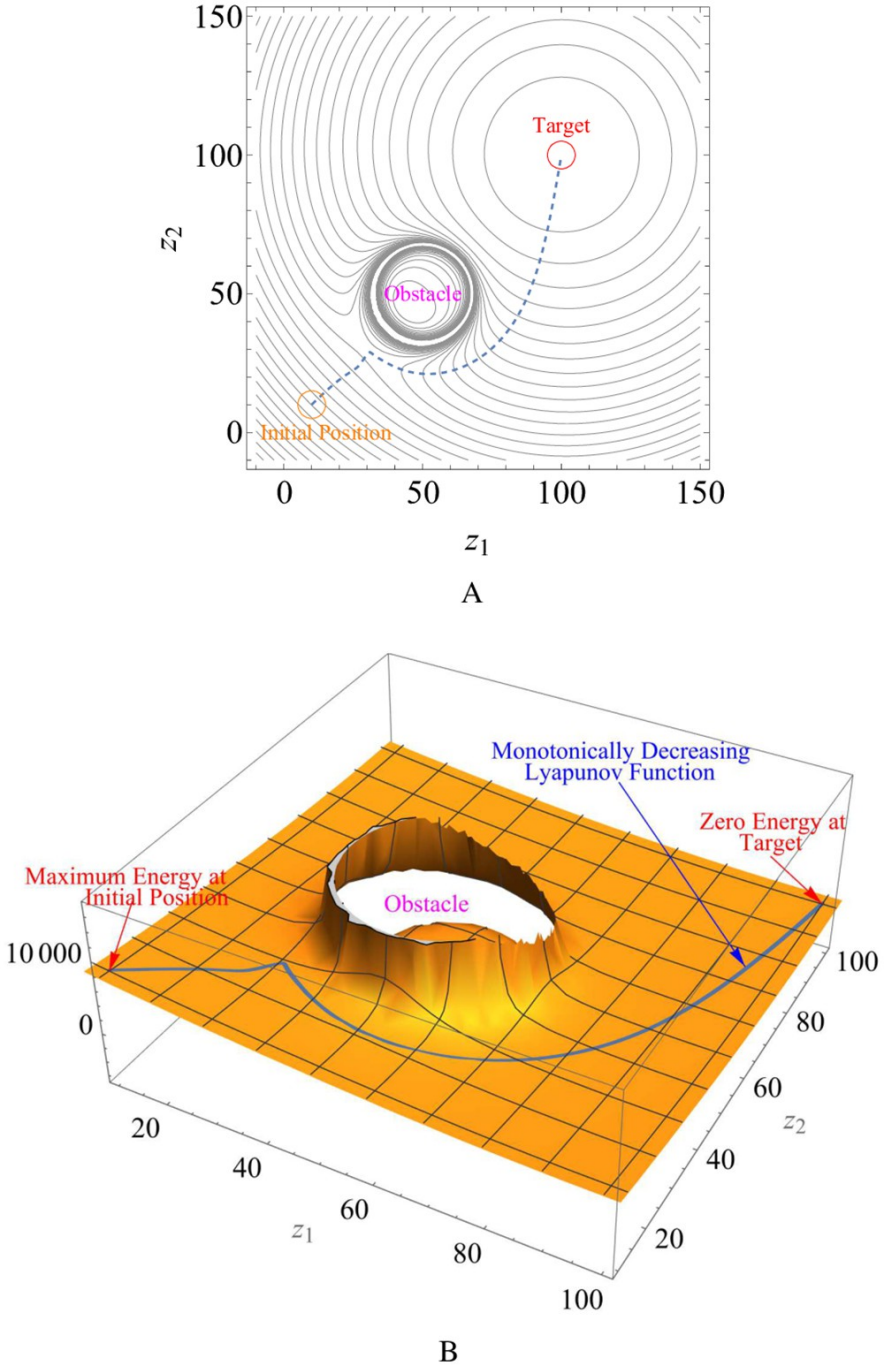
Evolution of the total potential seen through the attraction and repulsive potentials.

For instance, suppose a Lyapunov function $L=V+\frac{\alpha V}{W}$, where $V=\frac{1}{2}\left[{\left(x-100\right)}^{2}+{\left(y-100\right)}^{2}\right]$ is an attraction function, *α* = 1 is the tuning parameter and $W=\frac{1}{2}\left[{\left(x-55\right)}^{2}+{\left(y-60\right)}^{2}-10^{2}\right]$ is the obstacle avoidance function. The contour plot for a robot initially positioned at (10, 10) generated over a workspace −10 < *z*_1_ < 150 and −10 < *z*_2_ < 150 is shown in Fig 2A. The robot’s trajectory from the initial configuration to the target configuration is shown in a dashed line that avoids the obstacle at (55, 60). The 3D visualization of the repulsive and attractive potential fields is shown in Fig 2B. The evolution of the Lyapunov function is shown in a blue line, displaying that the robot’s energy is monotonically decreasing and zero at the target configuration.

## 4 System modeling

**Fig 3:**
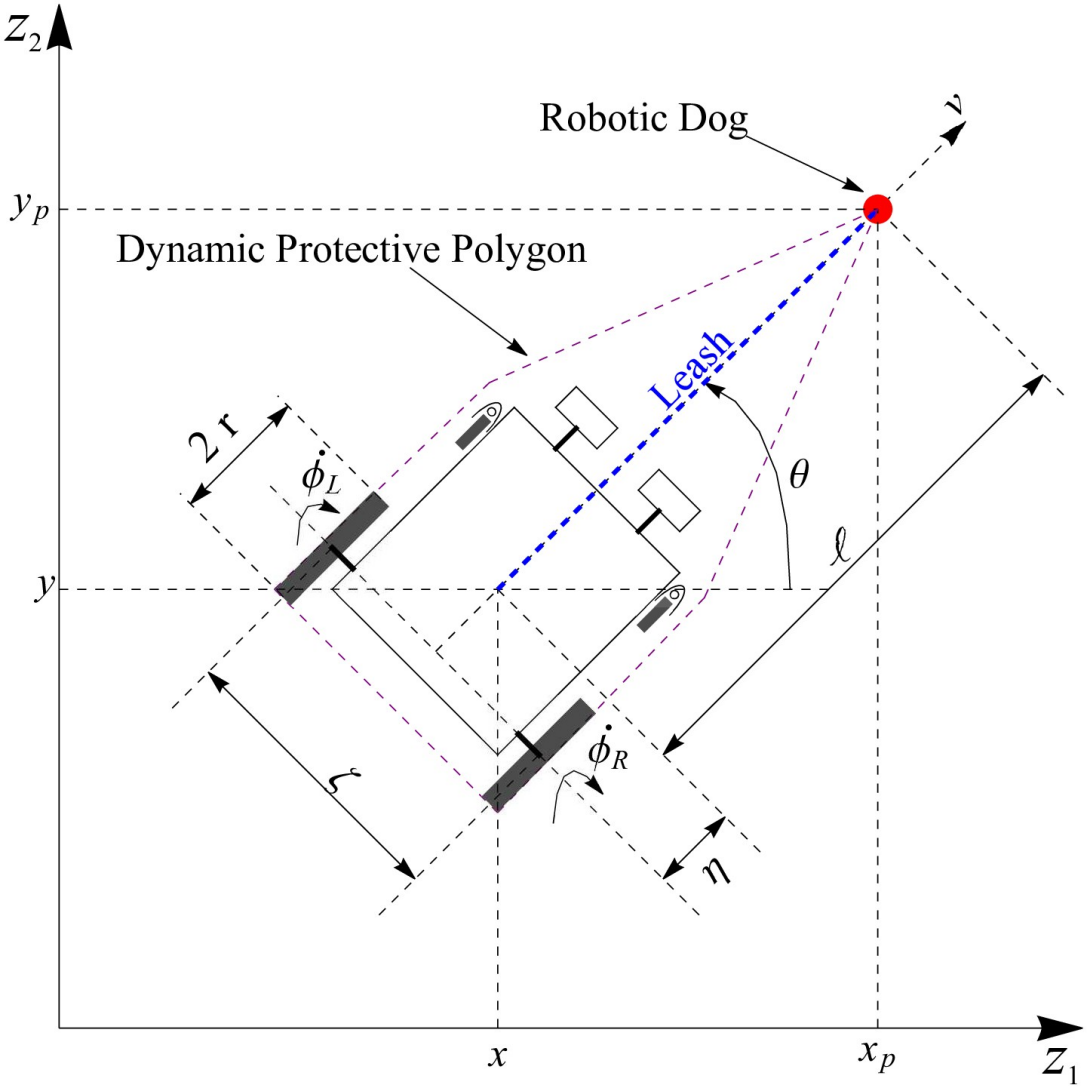
Schematic representation of a two rear-wheel driven wheelchair leashed to a robot dog.

Consider a planar two rear-wheel driven wheelchair robot with two front castor wheels as shown in Fig 3. The robot’s center of mass is at the cartesian coordinate (*x*, *y*). On opposing ends of a wheelbase, which of length *ζ*, are the two rear drive wheels of radius *r*. The robot’s orientation concerning *z*_1_- axis is given by *θ*. The center of the robot is at a length of *η* with orientation *θ* from the center of the two rear opposed wheels. The angular velocities of the rear left and right wheels are denoted as ${\dot{\phi }}_{L}=v_{L}$ and ${\dot{\phi }}_{R}=v_{R}$, respectively. To mobilize the wheelchair robot, it is connected to a robotic dog represented as a point-mass, positioned at (*x*_*p*_, *y*_*p*_), via a virtual leash of length *ℓ*, as shown in Fig 3. The role of the robotic dog is to guide the motion of the wheelchair. Essentially, this system observes the snow plough analogy from [11], where a collision and obstacle avoidance free path for the wheelchair is established by the robotic dog. The robotic dog is modelled as a moving point described in Definition 4.1.

**Definition 4.1** *Let P be a* point-based robotic dog *in the z*_1_*z*_2_ *plane, positioned at* (*x*_*p*_, *y*_*p*_) *moving with a velocity of v at time t* ≥ 0. *The robotic dog is a set*

$$P=\{\left(z_{1},z_{2}\right)\in R^{2}:{\left(z_{1}-x\right)}^{2}+{\left(z_{2}-y\right)}^{2}=0\}.$$

To obtain a kinodynamic model of the autonomous robotic dog wheelchair system, we accommodate the system’s dynamics by including its acceleration components. The acceleration-based control laws will permit motion control at higher speeds, while the kinematic models will lessen the effectiveness of the results at low speeds. Therefore, design

$$\begin{array}{l} {\dot{x}}_{p}=u_{1},\;\;{\dot{y}}_{p}=u_{2},\\ \dot{x}=\frac{r}{2}(v_{R}+v_{L})\;\text{cos}\;\theta +\frac{r\eta }{\zeta }(v_{L}-v_{R})\;\text{sin}\;\theta ),\\ \dot{y}=\frac{r}{2}(v_{R}+v_{L})\;\text{sin}\;\theta +\frac{r\eta }{\zeta }(v_{R}-v_{L})\;\text{cos}\;\theta ),\\ \dot{\theta }=\frac{r}{\zeta }(v_{R}-v_{L}),\\ {\dot{u}}_{1}=\rho_{1},\;\;\;{\dot{u}}_{2}=\rho_{2},\\ {\dot{v}}_{R}=\sigma_{1},\;\;\;{\dot{v}}_{L}=\sigma_{2}, \end{array}\}$$ (1)

where *u*_1_ and *u*_2_ are the *z*_1_ and *z*_2_ components, respectively, of *v*, and where *ρ*_*i*_ and *σ*_*i*_, *i* = 1, 2 are categorized as the nonlinear acceleration-based controllers. The vector notation $x=\left(x_{p},y_{p},x,y,\theta ,u_{1},u_{2},v_{R},v_{L}\right)\in R^{9}$ is used to refer to the velocities and positions of the different components of the autonomous robotic dog wheelchair system. In particular, let **x**_*a*_(*t*) = (*x*(*t*), *y*(*t*)) and **x**_0_(*t*) = (*x*_*p*_(*t*), *y*_*p*_(*t*)), refer to the rectangular position of wheelchair robot and robotic dog, respectively.

## 5 Motion planning and control

**Fig 4:**
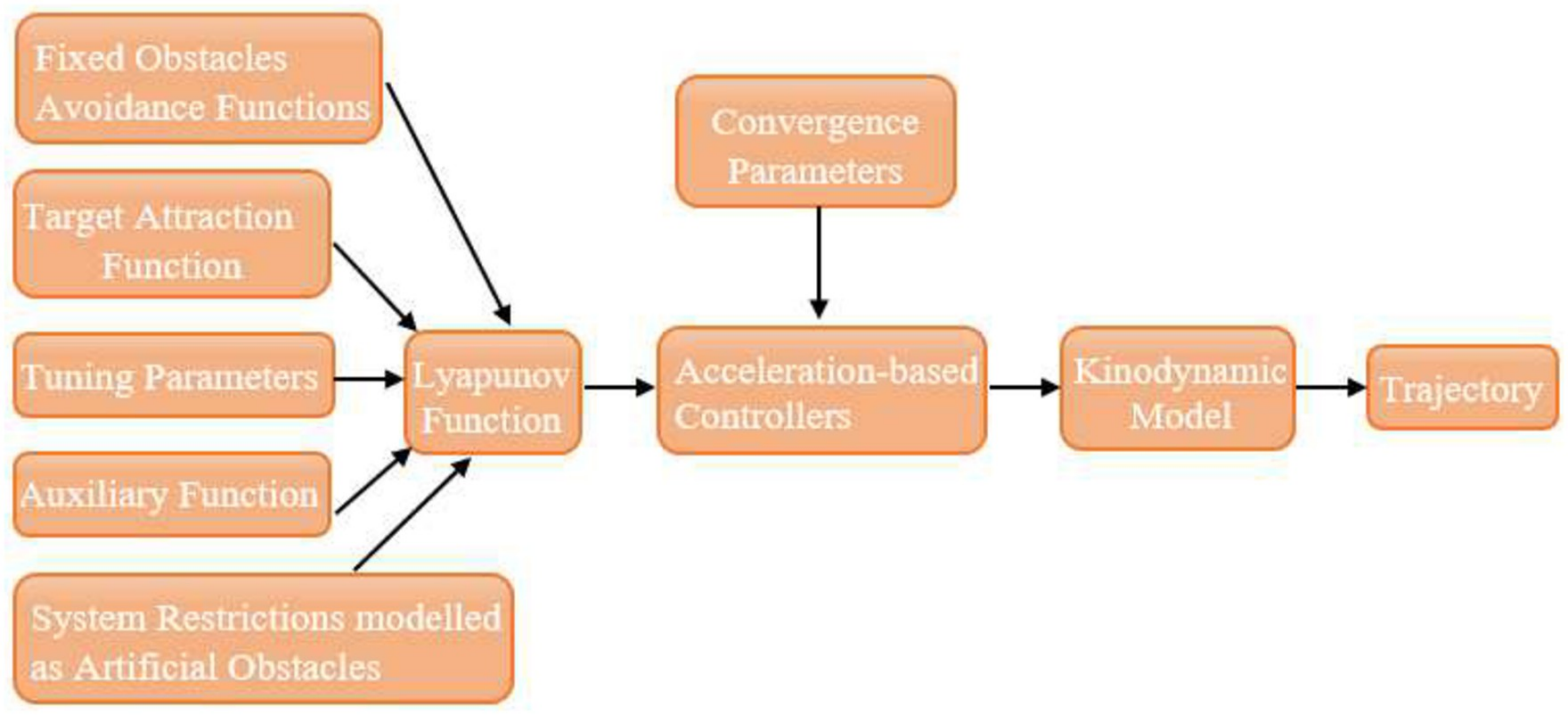
Block diagram exemplifying the Lyapunov-based control scheme.

The primary intent is to utilize the LbCS to derive the acceleration-based controllers (*ρ*_*i*_ and *σ*_*i*_, *i* = 1, 2,) for the autonomous robotic dog wheelchair system. The design of the control laws is represented in Fig 4.

### 5.1 Target attraction

Assign a target for the robotic dog to arrive after some time *t* > 0, enabling the user to reach the pre-determined target via the wheelchair system. This information can be fed to the robotic dog system by a pre-programmed centralized unit in-charge of the daily routine of the particular user. The target is a disk with radius *r*_*T*_ and center (*τ*_1_, *τ*_2_) defined as

$$\begin{array}{c} T=\{\left(z_{1},z_{2}\right)\in R^{3}:{\left\|\left(z_{1},z_{2}\right)-\left(\tau_{1},\tau_{2}\right)\right\|}^{2}\leq r_{T}^{2}\}. \end{array}$$

The following attractive potential function is established for target attraction

$$\begin{array}{c} V\left(x\right)=\frac{1}{2}[{\left(x_{p}-\tau_{1}\right)}^{2}+{\left(y_{p}-\tau_{2}\right)}^{2}+u_{1}^{2}+u_{2}^{2}+v_{R}^{2}+v_{L}^{2}]. \end{array}$$ (2)

### 5.2 Avoidance of obstacles

In line with the snowplough analogy designed in [11], herein an obstacle-free path will be traced by a dynamic polygonal protective region (see Fig 3) when the robotic dog avoids the fixed obstacles on its way. The wheelchair will traverse this obstacle-free path. The control laws for the wheelchair will only need to reflect the artificial obstacles and the system constraints. Therefore, the following types of obstacles are considered in this research.

1. Artificial obstacles—these are the singularities associated with the mechanical system. In particular, the bounds on velocities of wheelchair wheels and the robotic dog [31].
2. Fixed obstacles—this research considers a priori known workspace cluttered with stationary m∈N line obstacles and q∈N elliptic obstacles. The robotic dog and the dynamical polygon must avoid static obstacles while navigating the workspace.

#### 5.2.1 Artificial obstacles

To maneuver in reality, for safety reasons, the velocities of the autonomous robotic dog wheelchair system must be bounded. Thus the following restrictions are imposed:

- |*u*_*i*_(*t*)| ≤ *u*_max_ (for *i* = 1, 2);
- |*v*_*R*_(*t*)| ≤ *v*_max_, and
- |*v*_*L*_(*t*)| ≤ *v*_max_,

where *v*_max_ > 0 is the maximum velocity and kept the same for the robotic dog and wheelchair. The following potential functions will ensure restrictions on the velocities

$$\begin{array}{cc} \;S_{1}\left(x\right)=\frac{1}{2}(u_{\text{max}}^{2}-u_{1}^{2})\;, & S_{2}\left(x\right)=\frac{1}{2}(u_{\text{max}}^{2}-u_{2}^{2})\;, \end{array}$$ (3a-b)

$$\begin{array}{cc} S_{3}\left(x\right)=\frac{1}{2}(v_{\text{max}}^{2}-v_{R}^{2})\;, & S_{4}\left(x\right)=\frac{1}{2}(v_{\text{max}}^{2}-v_{L}^{2})\;. \end{array}$$ (3c-d)

Later, in Section 6 the functions constructed above will be added as a ratio to the total potentials of the system.

#### 5.2.2 Fixed elliptic obstacles

Let the workspace of the system (1) contain q∈N elliptic obstacles of random sizes and positions. The purpose of using elliptic obstacles is that most 2-dimensional objects can be inscribed within an ellipse with minimum overlap.

**Definition 5.1** *The lth elliptic obstacle is centered at* (*o*_*l*1_, *o*_*l*2_) *with width a*_*l*_ > 0 *and height b*_*l*_ > 0 *on the* *z*_1_*z*_2_ *plane is described as*

$$FO_{l}=\{\left(z_{1},z_{2}\right)\in R^{2}:\frac{{\left(z_{1}-o_{l1}\right)}^{2}}{a_{l}^{2}}+\frac{{\left(z_{2}-o_{l2}\right)}^{2}}{b_{l}^{2}}\leq 1\},$$

*for l* = 1, 2, …, *q*.

Following the snowplough analogy [11], we enclose the autonomous robotic dog wheelchair system in a polygonal protective region as shown in Fig 3. This polygonal protective region will trace a obstacle free path for the robotic dog wheelchair system. To generate a collision free path, it is important that every point on the boundary of the polygonal protective region must avoid the obstacles. The minimum distance technique (MDT) proposed by Sharma in [31] is utilized for this. The fundamental concept is to locate a point on the boundary of the polygonal protective region that is nearest to an obstacle and at any time *t* ≥ 0 the nearest point (and hence the entire polygon) will avoid the obstacle. This basically means that led by the robotic dog the wheelchair will be in a path that is obstacle free.

For the avoidance of the elliptical obstacles by the polygonal protective region, the following potential functions are constructed

$$\begin{array}{c} W_{jl}\left(x\right)=\frac{{\left(x_{j}^{*}-2\lambda_{l}\left(\eta +r\right)\;\text{cos}\;\theta -o_{l1}\right)}^{2}}{2a_{l}^{2}}+\frac{{\left(y_{j}^{*}-2\lambda_{l}\left(\eta +r\right)\;\text{sin}\;\theta -o_{l2}\right)}^{2}}{2b_{l}^{2}}-\frac{1}{2},\; \end{array}$$ (4a)

$$\begin{array}{c} W_{jl}^{*}\left(x\right)=\frac{{\left(x_{j}^{*}+\lambda_{jl}^{*}(x_{p}-x_{j}^{*})-o_{l1}\right)}^{2}}{2a_{l}^{2}}+\frac{{\left(y_{j}^{*}+\lambda_{jl}^{*}(y_{p}-y_{j}^{*})-o_{l2}\right)}^{2}}{2b_{l}^{2}}-\frac{1}{2},\; \end{array}$$ (4b)

where

$$\begin{array}{ccc} x_{j}^{*} & = & x+\left(\eta +r\right)\;\text{cos}\;\theta +\frac{\zeta }{2}{\left(-1\right)}^{j}\;\text{sin}\;\theta ,\\ y_{j}^{*} & = & y+\left(\eta +r\right)\;\text{sin}\;\theta -\frac{\zeta }{2}{\left(-1\right)}^{j}\;\text{cos}\;\theta ,\\ \lambda_{l} & = & \text{min}\;\{\text{max}\;\{0,\chi \},1\},\\ \lambda_{jl}^{*} & = & \text{min}\;\{\text{max}\;\{0,\varrho \},1\}, \end{array}$$

with

$$\begin{array}{c} \chi =\frac{b_{l}^{2}\left(x_{j}^{*}-o_{l1}\right)\;\text{cos}\;\theta +a_{l}^{2}\left(y_{j}^{*}-o_{l2}\right)\;\text{sin}\;\theta }{2\left(r+\eta \right)\left(b_{l}^{2}\;{\text{cos}}^{2}\theta +a_{l}^{2}\;{\text{sin}}^{2}\theta \right)} \end{array}$$

and

$$\begin{array}{c} \varrho =\frac{b_{l}^{2}\left(x_{j}^{*}-o_{l1}\right)\left(x_{j}^{*}-x_{p}\right)+a_{l}^{2}\left(y_{j}^{*}-o_{l2}\right)\left(y_{j}^{*}-y_{p}\right)}{b_{l}^{2}{\left(x_{j}^{*}-x_{p}\right)}^{2}+a_{l}^{2}{\left(y_{j}^{*}-y_{p}\right)}^{2}} \end{array}$$

for *l* = 1, 2, …, *q* and *j* = 1, 2.

#### 5.2.3 Fixed line obstacles

Consider the workspace of system (1) cluttered with *m* > 0 line obstacles. The *k*th line obstacle is defined as:

**Definition 5.2** *The kth line segment in the z*_1_*z*_2_ *plane, from point* (*a*_*k*1_, *b*_*k*1_) *to point* (*a*_*k*2_, *b*_*k*2_) *is the set*

$$\begin{array}{ccc} LO_{k} & = & \{\left(z_{1},z_{2}\right)\in R^{2}:{\left(z_{1}-a_{k1}-\beta_{k}\left(a_{k2}-a_{k1}\right)\right)}^{2}\\ & & \;\;\;+{\left(z_{2}-b_{k1}-\beta_{k}\left(b_{k2}-b_{k1}\right)\right)}^{2}=0\}, \end{array}$$

*where β*_*k*_ ∈ [0, 1], *k* = 1, 2, …, *m*.

Utilizing the MDT, the following functions are constructed for the avoidance of the line obstacles by the polygonal protective region:

$$\begin{array}{cc} R_{jk}\left(x\right) & =\frac{1}{2}[{\left(x_{j}^{*}-2\gamma_{jk}\left(\eta +r\right)\;\text{cos}\;\theta -a_{k1}-\beta_{jk}\left(a_{k2}-a_{k1}\right)\right)}^{2}\\ & \;+{\left(y_{j}^{*}-2\gamma_{jk}\left(\eta +r\right)\;\text{sin}\;\theta -b_{k1}-\beta_{jk}(b_{k2}-b_{k1}\right)}^{2}], \end{array}$$ (5a)

$$\begin{array}{cc} R_{jk}^{*}\left(x\right) & =\frac{1}{2}[{\left(x_{j}^{*}+\gamma_{jl}^{*}(x_{p}-x_{j}^{*})-a_{k1}-\beta_{jk}^{*}\left(a_{k2}-a_{k1}\right)\right)}^{2}\\ & \;+{\left(y_{j}^{*}+\gamma_{jl}^{*}(y_{p}-y_{j}^{*})-b_{k1}-\beta_{jk}^{*}(b_{k2}-b_{k1}\right)}^{2}],\; \end{array}$$ (5b)

where

$$\begin{array}{c} \beta_{jk}=\text{min}\;\{\text{max}\;\{0,\mu \},1\}\\ \beta_{jk}^{*}=\text{min}\;\{\text{max}\;\{0,\varpi \},1\}\\ \gamma_{jk}=\text{min}\;\{\text{max}\;\{0,\varepsilon \},1\}\\ \gamma_{jk}^{*}=\text{min}\;\{\text{max}\;\{0,\varsigma \},1\} \end{array}$$

with

$$\begin{array}{c} \mu =\frac{\left(b_{k1}-y_{j}^{*}\right)\;\text{cos}\;\theta +\left(x_{j}^{*}-a_{k1}\right)\;\text{sin}\;\theta }{\left(b_{k1}-b_{k2}\right)\;\text{cos}\;\theta +\left(a_{k2}-a_{k1}\right)\;\text{sin}\;\theta }, \end{array}$$

$$\begin{array}{c} \varpi =\frac{b_{k1}\left(x_{p}-x_{j}^{*}\right)-x_{p}y_{j}^{*}+a_{k1}\left(y_{j}^{*}-y_{p}\right)+x_{j}^{*}y_{p}}{\left(a_{k1}-a_{k2}\right)\left(y_{j}^{*}-y_{p}\right)-\left(b_{k1}-b_{k2}\right)\left(x_{j}^{*}-x_{p}\right)}, \end{array}$$

$$\begin{array}{c} \varepsilon =\frac{\left(b_{k1}-b_{k2}\right)x_{j}^{*}+a_{k1}\left(b_{k2}-y_{j}^{*}\right)+a_{k2}\left(y_{j}^{*}-b_{k1}\right)}{2\left(\eta +r\right)(\left(b_{k1}-b_{k2}\right)\;\text{cos}\;\theta +\left(a_{k2}-a_{k1}\right)\;\text{sin}\;\theta )}, \end{array}$$

and

$$\begin{array}{c} \varsigma =\frac{\left(b_{k1}-b_{k2}\right)x_{j}^{*}+a_{k1}\left(b_{k2}-y_{j}^{*}\right)+a_{k2}\left(y_{j}^{*}-b_{k1}\right)}{\left(b_{k1}-b_{k2}\right)\left(x_{j}^{*}-x_{p}\right)-\left(a_{k1}-a_{k2}\right)\left(y_{j}^{*}-y_{p}\right)} \end{array}$$

for *k* = 1, 2, …, *m* and *j* = 1, 2.

#### 5.2.4 Inter-robot bound

For the wheel chair robot to efficiently follow the point-based robotic dog, it is important that the distance between the two robots should not exceed *ℓ*, the leash length as shown in Fig 3, and be no less than *ℓ*_*min*_ so that they do not collide. That is, ||**x**_0_(*t*) − **x**_*a*_(*t*)|| ≤ *ℓ* and ||**x**_0_(*t*) − **x**_*a*_(*t*)|| ≥ *ℓ*_*min*_. To adhere to these restrictions, the subsequent avoidance functions are proposed

$$\begin{array}{ccc} A_{1}\left(x\right) & = & \frac{1}{2}[\ell^{2}-{\left(x-x_{p}\right)}^{2}-{\left(y-y_{p}\right)}^{2}] \end{array}$$ (6)

$$\begin{array}{ccc} A_{2}\left(x\right) & = & \frac{1}{2}[{\left(x-x_{p}\right)}^{2}+{\left(y-y_{p}\right)}^{2}-\ell_{min}^{2}]. \end{array}$$ (7)

## 6 Design of control laws

A total potential for system (1) given by

$$\begin{array}{ccc} L(x) & = & V\left(x\right)+F\left(x\right)[\sum_{i=1}^{2}\frac{\alpha_{i}}{A_{i}\left(x\right)}+\sum_{i=1}^{4}\frac{\xi_{i}}{S_{i}\left(x\right)}+\sum_{j=1}^{2}\sum_{l=1}^{q}(\frac{\beta_{jl}}{W_{jl}\left(x\right)}+\frac{\beta_{jl}^{*}}{W_{jl}^{*}\left(x\right)})\\ & & +\sum_{j=1}^{2}\sum_{k=1}^{m}(\frac{\varphi_{jk}}{R_{jk}\left(x\right)}+\frac{\varphi_{jk}^{*}}{R_{jk}^{*}\left(x\right)})], \end{array}$$ (8)

which is obtained by combining all the potential functions (2)–(7) and introducing *control parameters*, *α*_*i*_ > 0, *ξ*_*i*_ > 0, *β*_*jl*_ > 0, $\beta_{jl}^{*}>0$, *φ*_*jk*_ > 0 and $\varphi_{jk}^{*}>0$ where i,j,k,l∈N.

Additionally, the auxiliary function

$$\begin{array}{c} F\left(x\right)=\frac{1}{2}[{\left(x_{p}-\tau_{1}\right)}^{2}+{\left(y_{p}-\tau_{2}\right)}^{2}] \end{array}$$

in Eq (8) is necessary for the Lyapunov function to attain a value of zero at the target. Over the domain

$$\begin{array}{ccc} D & = & \{x\in R^{9}:\;A_{n}\left(x\right)>0,\;S_{i}\left(x\right)>0,\;W_{jl}\left(x\right)>0,\;W_{jl}^{*}\left(x\right)>0,\;R_{jk}\left(x\right)>0,\\ & & \;R_{jk}^{*}\left(x\right)>0,\;n=1,2,\;i=1,2,3,4,\;j=1,2,\;k=1,2,\ldots ,m,\;l=1,2,\ldots ,q\}, \end{array}$$

the Lyapunov function (8) is positive, continuous, and has a bound. Suppose θ* is the orientation of the wheelchair at the target, then the point **e** = (*τ*_1_, *τ*_2_, *τ*_1_ − *a*, *τ*_2_ − *b*, θ*, 0, 0, 0, 0) is an equilibrium point of system (1). The control laws designed such that **e** is at least a stable equilibrium point are captured in Theorem 6.1.

**Theorem 6.1** *The equilibrium point* **e** = (*τ*_1_, *τ*_2_, *τ*_1_ − *a*, *τ*_2_ − *b*, θ*, 0, 0, 0, 0) *of system* (1) *is stable provided the controllers ρ*_*i*_ *and* *σ*_*i*_ (*i* = 1, 2) *are defined as*

$$\begin{array}{c} \begin{array}{cc} \rho_{1}= & -(\delta_{1}u_{1}+\frac{\partial L}{\partial x_{p}})/(1+\frac{\alpha_{1}F}{S_{1}^{2}})\;,\\ \rho_{2}= & -(\delta_{2}u_{2}+\frac{\partial L}{\partial y_{p}})/(1+\frac{\alpha_{2}F}{S_{2}^{2}})\;,\\ \sigma_{1}= & -(\delta_{3}v_{R}+\frac{\partial L}{\partial x}[\frac{r}{2}\;\text{cos}\;\theta -\frac{r\eta }{\zeta }\;\text{sin}\;\theta ]\\ & +\frac{\partial L}{\partial y}[\frac{r}{2}\;\text{sin}\;\theta +\frac{r\eta }{\zeta }\;\text{cos}\;\theta ]+\frac{r}{\zeta }\frac{\partial L}{\partial \theta })/(1+\frac{\alpha_{3}F}{S_{3}^{2}})\;,\\ \sigma_{2}= & -(\delta_{4}v_{L}+\frac{\partial L}{\partial x}[\frac{r}{2}\;\text{cos}\;\theta +\frac{r\eta }{\zeta }\;\text{sin}\;\theta ]\\ & +\frac{\partial L}{\partial y}[\frac{r}{2}\;\text{sin}\;\theta -\frac{r\eta }{\zeta }\;\text{cos}\;\theta ]-\frac{r}{\zeta }\frac{\partial L}{\partial \theta })/(1+\frac{\alpha_{4}F}{S_{4}^{2}})\;, \end{array}\} \end{array}$$ (9)

*where* *δ*_*i*_ > 0, (*i* = 1, 2, 3, 4) *are called the convergence parameters*.

**Proof**: It should be noted that the Lyapunov function *L*(**x**) specified in (8) is both continuous and bounded, with a positive value throughout the domain *D*. Additionally, *L*(**e**) = 0 and *L*(**x**) > 0 for any **x** ≠ **e** within the domain *D*. Furthermore, the first partial derivatives of *L*(**x**) are continuous within the domain *D* and therefore

$$\begin{array}{ccc} \dot{L}\left(x\right) & = & \frac{\partial L}{\partial x_{p}}{\dot{x}}_{p}+\frac{\partial L}{\partial y_{p}}{\dot{y}}_{p}+\frac{\partial L}{\partial x}\dot{x}+\frac{\partial L}{\partial y}\dot{y}+\frac{\partial L}{\partial \theta }\dot{\theta }+\frac{\partial L}{\partial u_{1}}{\dot{u}}_{1}+\frac{\partial L}{\partial u_{2}}{\dot{u}}_{2}+\frac{\partial L}{\partial v_{R}}{\dot{v}}_{R}+\frac{\partial L}{\partial v_{L}}{\dot{v}}_{L}\\ & = & \frac{\partial L}{\partial x_{p}}u_{1}+\frac{\partial L}{\partial y_{p}}u_{2}+\frac{\partial L}{\partial \theta }\frac{r}{\zeta }\left(v_{R}-v_{L}\right)\\ & & +\frac{\partial L}{\partial x}(\frac{r}{2}\left(v_{R}+v_{L}\right)\;\text{cos}\;\theta +\frac{r\eta }{\zeta }\left(v_{L}-v_{R}\right)\;\text{sin}\;\theta )\\ & & +\frac{\partial L}{\partial y}(\frac{r}{2}\left(v_{R}+v_{L}\right)\;\text{sin}\;\theta +\frac{r\eta }{\zeta }\left(v_{R}-v_{L}\right)\;\text{cos}\;\theta )\\ & & +(1+\frac{\alpha_{1}F}{S_{1}^{2}})u_{1}\rho_{1}+(1+\frac{\alpha_{2}F}{S_{2}^{2}})u_{2}\rho_{2}\\ & & +(1+\frac{\alpha_{1}F}{S_{3}^{2}})v_{R}\sigma_{1}+(1+\frac{\alpha_{1}F}{S_{4}^{2}})v_{L}\sigma_{2}\\ & = & [\frac{\partial L}{\partial x_{p}}+\rho_{1}(1+\frac{\alpha_{1}F}{S_{1}^{2}})]u_{1}+[\frac{\partial L}{\partial y_{p}}+\rho_{2}(1+\frac{\alpha_{2}F}{S_{2}^{2}})]u_{2}+\Omega v_{R}+\Psi v_{L} \end{array}$$

where

$$\begin{array}{ccc} \Omega & = & \frac{\partial L}{\partial x}(\frac{r}{2}\;\text{cos}\;\theta -\frac{r\eta }{\zeta }\;\text{sin}\;\theta )+\sigma_{1}(1+\frac{\alpha_{3}F}{S_{3}^{2}})+\frac{\partial L}{\partial y}(\frac{r}{2}\;\text{sin}\;\theta +\frac{r\eta }{\zeta }\;\text{cos}\;\theta )+\frac{r}{\zeta }\frac{\partial L}{\partial \theta } \end{array}$$

and

$$\begin{array}{ccc} \Psi & = & \frac{\partial L}{\partial x}(\frac{r}{2}\;\text{cos}\;\theta +\frac{r\eta }{\zeta }\;\text{sin}\;\theta )+\sigma_{2}(1+\frac{\alpha_{4}F}{S_{4}^{2}})+\frac{\partial L}{\partial y}(\frac{r}{2}\;\text{sin}\;\theta -\frac{r\eta }{\zeta }\;\text{cos}\;\theta )-\frac{r}{\zeta }\frac{\partial L}{\partial \theta }. \end{array}$$

Substituting *ρ*_1_, *ρ*_2_, *σ*_1_ and *σ*_2_ from (9), we obtain

$$\begin{array}{c} \dot{L}\left(x\right)=-\delta_{1}u_{1}^{2}-\delta_{2}u_{2}^{2}-\delta_{3}v_{L}^{2}-\delta_{4}v_{R}^{2}\leq 0. \end{array}$$

The inequality $\dot{L}\left(x\right)\leq 0$ and the equality $\dot{L}\left(e\right)=0$ imply the stability of equilibrium point **e** in system (1) over the domain *D*.

## 7 Computer simulations

In this section, we numerically verify the effectiveness of the control scheme through simulations. The simulation results shown in three scenarios mimic real-life application of the autonomous robotic dog wheelchair system.

### 7.1 Scenario 1

**Fig 5:**
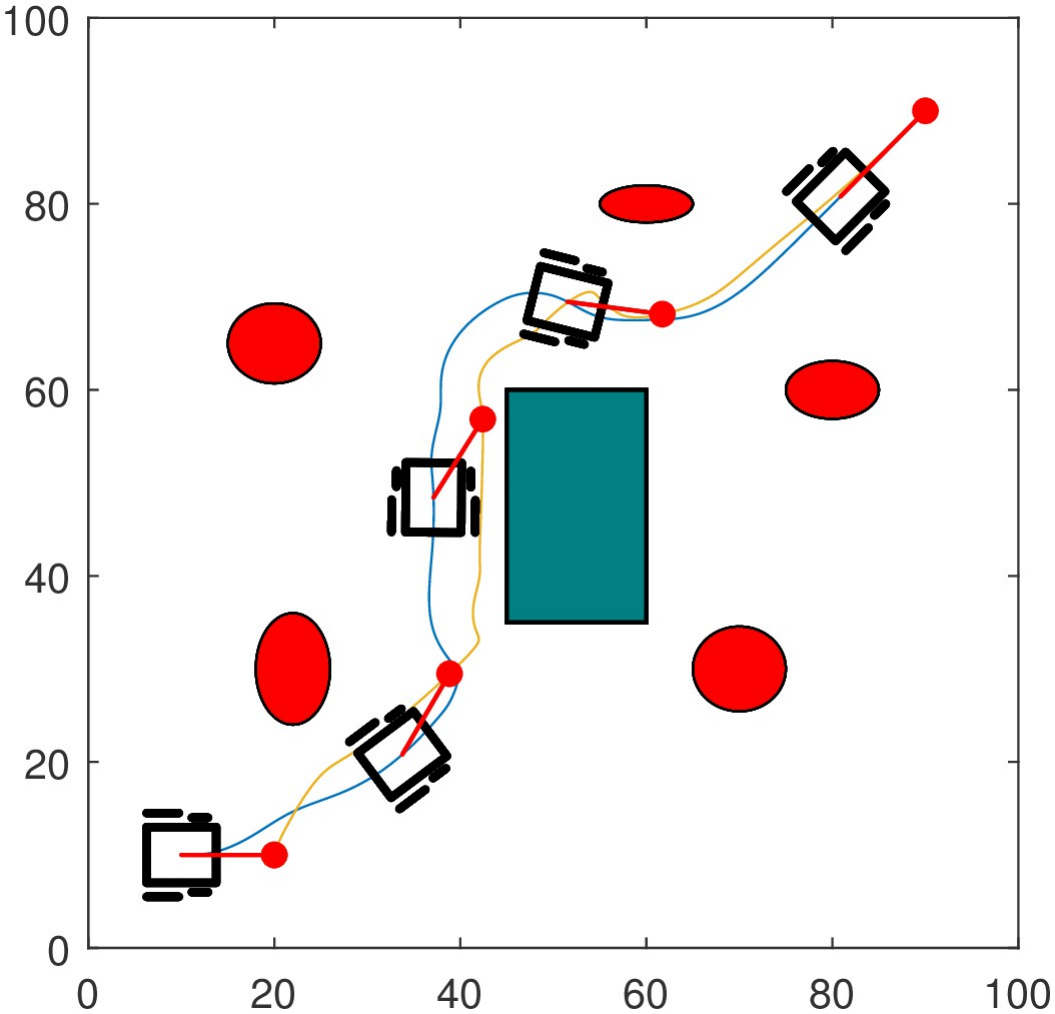
Path followed by the robotic dog (in yellow) and the wheelchair robot (in blue).

Consider a set-up as shown in Fig 5 where the robotic dog has to move from an initial position (20,10) to the target at (90,90) while guiding the wheelchair. The workspace contains five elliptic and one rectangular obstacles. This scenario resembles a park-like environment where the elliptic obstacles represent flower gardens or trees and the rectangular obstacle represents a building. Each edge of the rectangular obstacle is treated as a line obstacle. Fig 5 shows the path followed by the system and its convergence to a final configuration. The different parameters used in the simulation are given below.

**1. Robot parameters:**
  *r* = 1.75, *η* = 2.0, *ζ* = 6.
**2. Initial and final positions:**
  **x**_0_(0) = (20, 10), **x**_*a*_(0) = (10, 10), *θ*(0) = 0, (*τ*_1_, *τ*_2_) = (90, 90).
**3. Fixed elliptic obstacle:**
  Sizes and positions are random.
**4. Fixed line obstacle:**
  (*a*_11_, *b*_11_) = (45, 35), (*a*_21_, *b*_21_) = (60, 35), (*a*_12_, *b*_12_) = (45, 35), (*a*_22_, *b*_22_) = (45, 60), (*a*_13_, *b*_13_) = (45, 60), (*a*_23_, *b*_23_) = (60, 60), (*a*_14_, *b*_14_) = (60, 35), (*a*_24_, *b*_24_) = (60, 60).
**5. Physical limitations:**
  *v*_max_ = 5 *units*/*s* and *u*_max_ = 5 *units*/*s*.
**6. Convergence parameters:**
  *δ*_*i*_ = 10 for *i* = 1, …, 4.
**7. Control parameters:**
  *α* = 1, *ξ*_*i*_ = 0.01 for *i* = 1, …, 4, *β*_*jl*_ = 0.5, $\beta_{jl}^{*}=1$, *φ*_*jk*_ = 0.1, $\varphi_{jk}^{*}=0.1$ for *l* = 1, …, 5, *k* = 1, …, 4 and *j* = 1, 2,
**8. Workspace boundaries:**
  0 ≤ *z*_1_ ≤ 100 and 0 ≤ *z*_2_ ≤ 100.
**9. Other parameters:**
  *ℓ* = 13 units, *ℓ*_*min*_ = 10.

**Fig 6:**
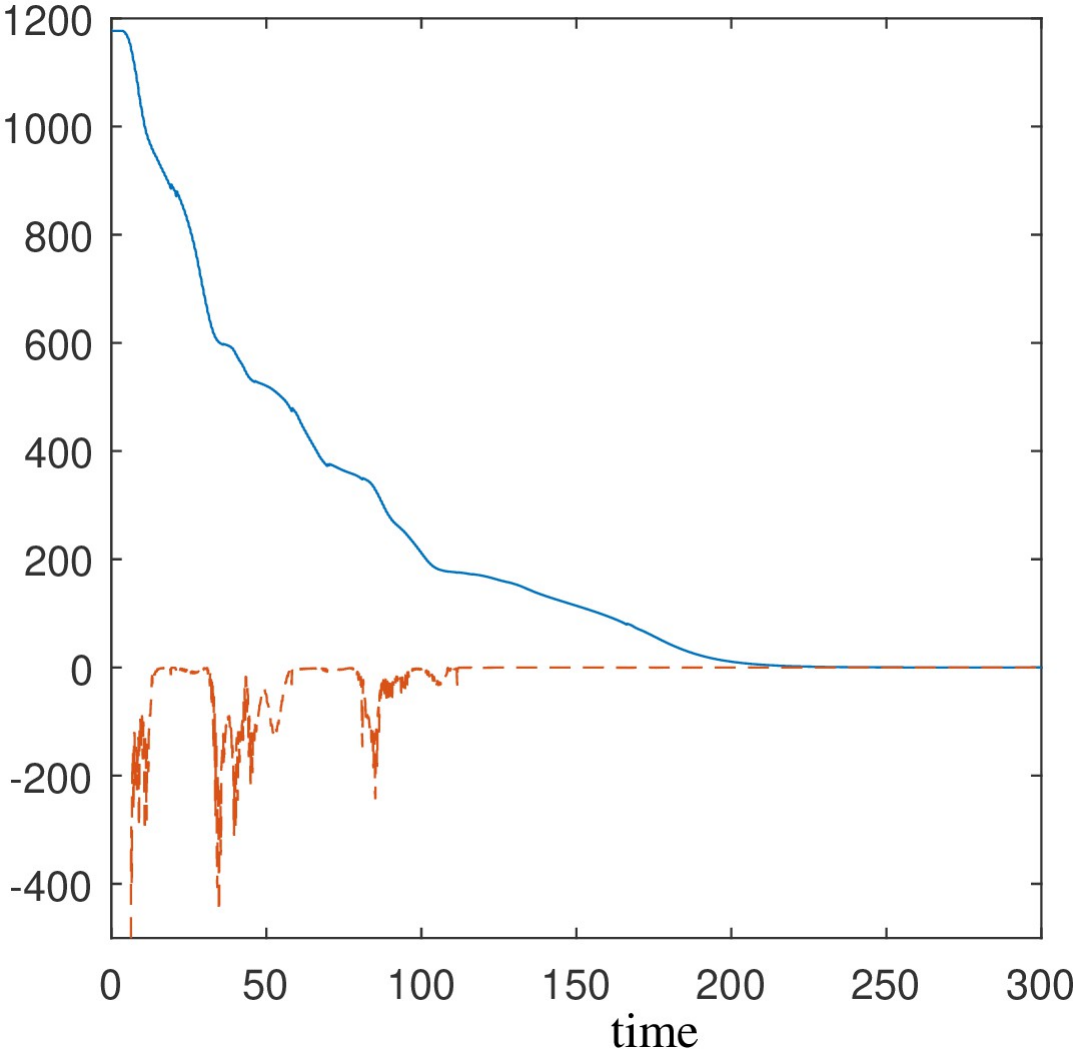
The trajectory depicted in Scenario 1 is accompanied by the evolution of *L*(x) (represented in blue and solid) and its time derivative $\dot{L}\left(\text{x}\right)$ (represented in red and dashed).

The trajectory of the system is accompanied by the evolution of both the Lyapunov function and its time derivative, as demonstrated in Fig 6. One can clearly notice the decreasing nature of the Lyapunov function which numerically verifies the stability property of the autonomous system.

**Fig 7:**
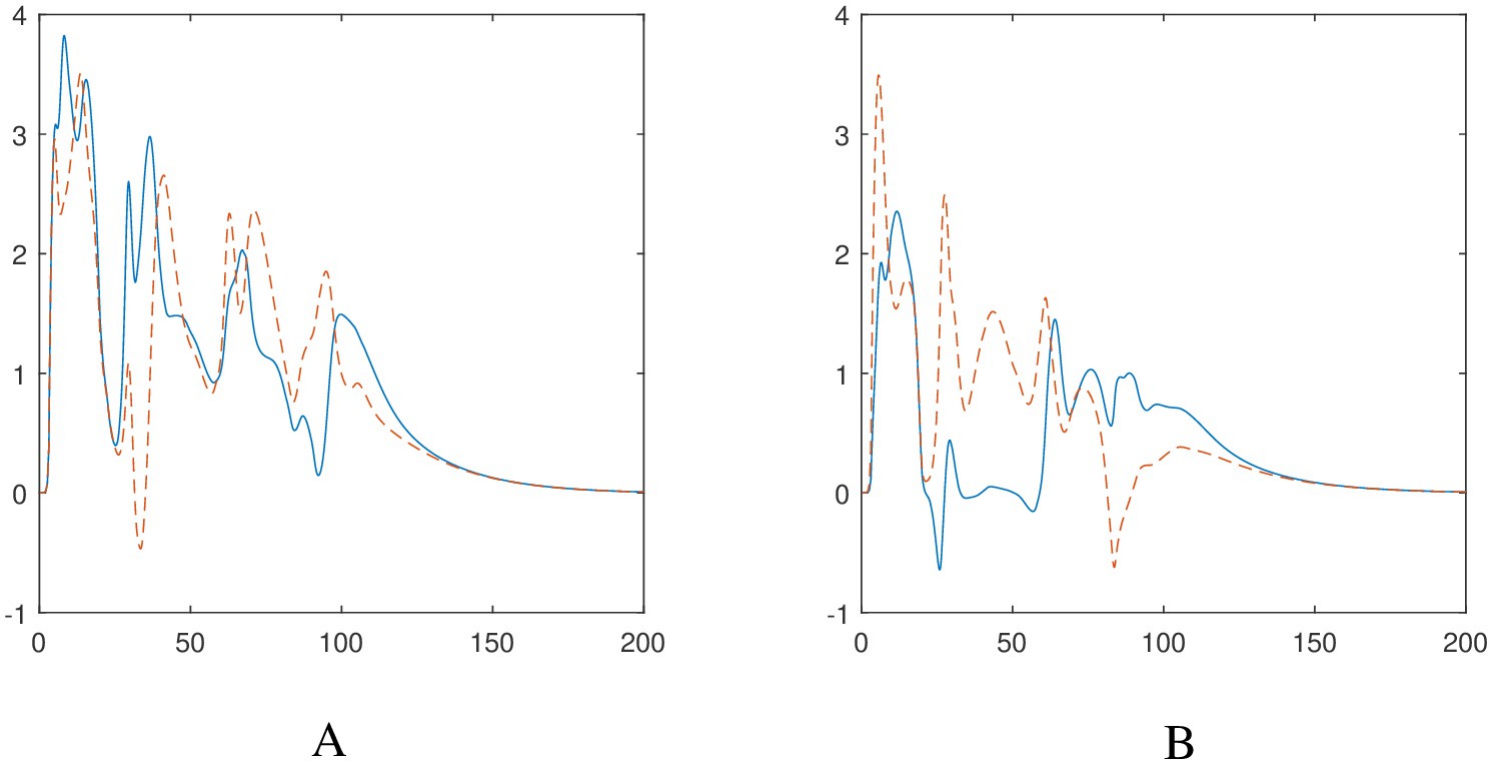
Evolution of nonlinear velocities for the autonomous robotic dog wheelchair system along the trajectory shown in Fig 5. A: Wheelchair velocities *v*_*R*_ in blue (solid), *v*_*L*_ in red (dashed). B: Robotic dog velocities *u*_1_ in blue (solid), *u*_2_ in red (dashed).

We have generated the graphs of velocities of the autonomous robotic dog wheelchair system to show where the robots have increased or decreased their speeds. Fig 7 shows the velocity profiles for the two robots and the asymptotic convergence of the velocities as *t* → ∞.

**Fig 8:**
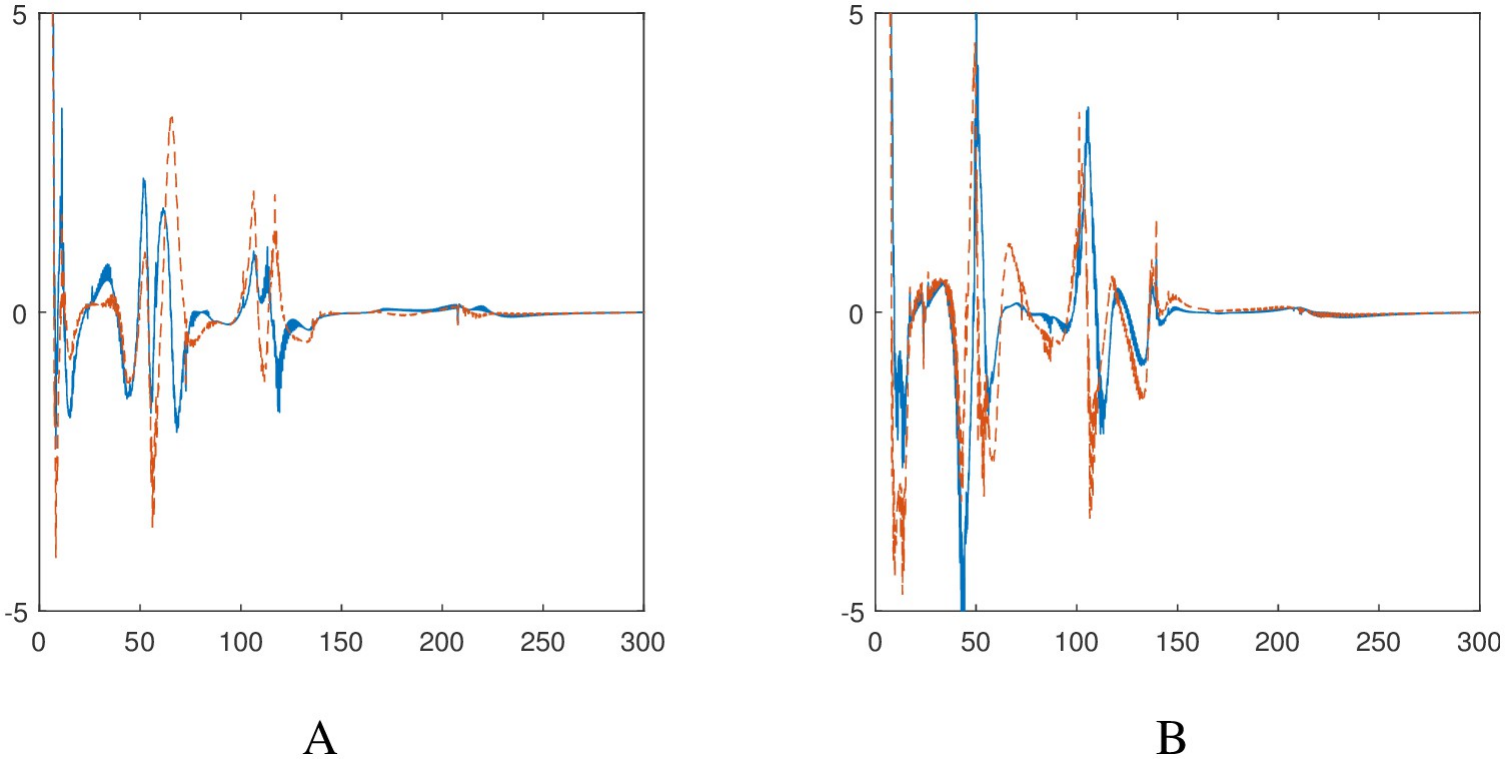
Evolution of the nonlinear controllers for the autonomous robotic dog wheelchair system along the trajectory shown in Scenario 1. A: Wheelchair controllers *σ*_1_ in blue (solid), *σ*_2_ in red (dashed). B: Robotic dog controllers *ρ*_1_ in blue (solid), *ρ*_2_ in red (dashed).

Fig 8 illustrates the behavior of the controllers during the trajectory of the two robots, and their convergence at the final state validates the efficacy of the proposed nonlinear acceleration-based controllers.

### 7.2 Scenario 2

**Fig 9:**
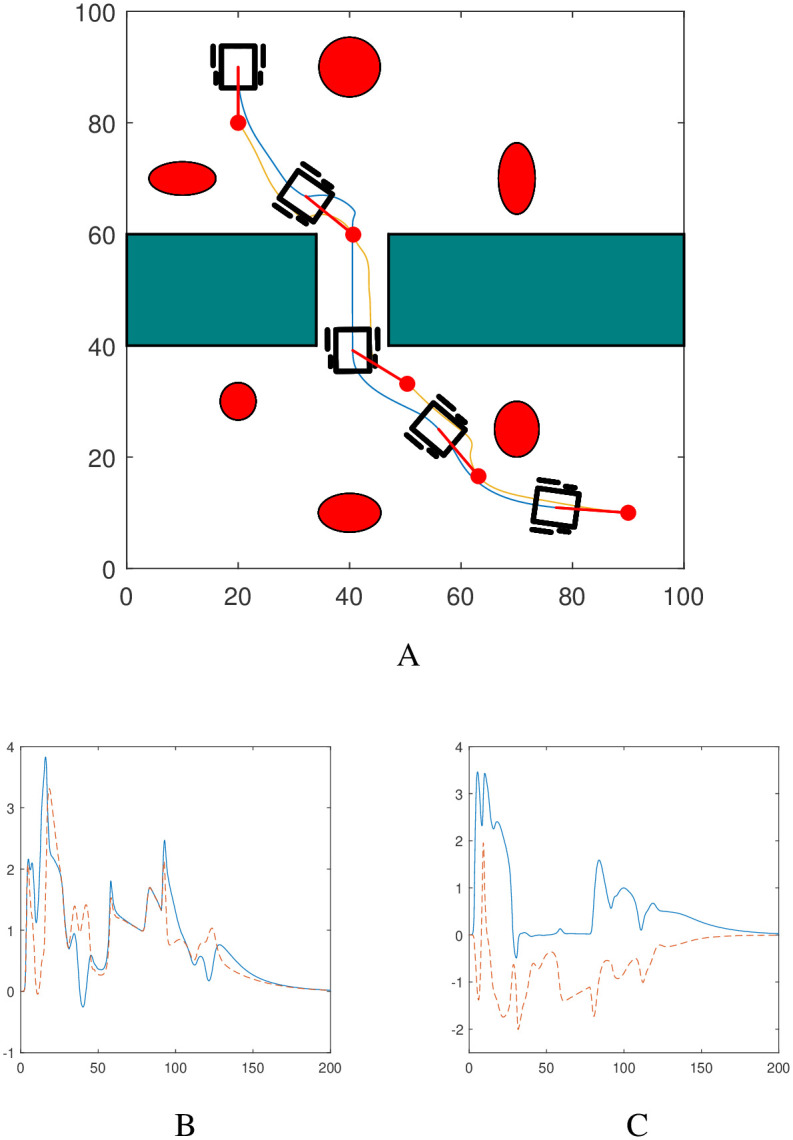
Evolution of trajectories of the robotic dog, wheelchair and the nonlinear velocities for the robotic dog wheelchair system along the trajectory shown in Scenario 2. A: Path followed by the robotic dog (in yellow) and the wheelchair robot (in blue). B: Velocities *v*_*R*_ in blue (solid), *v*_*L*_ in red (dashed). C: Velocities *u*_1_ in blue (solid), *u*_2_ in red (dashed).

The simulation result in Fig 9A show the robotic dog wheelchair system going through a narrow space in between two rectangular obstacles. This scenario mimics a narrow passage between two buildings or a narrow bridge. The workspace also contains elliptic obstacles of random sizes. The time evolution of the pertinent velocities during the autonomous system’s trajectory is explicitly depicted in Fig 9B and 9C. The asymptotic convergence of the velocities towards the final state is evident. Moreover, the evolution of the controllers and the Lyapunov function is akin to that shown in Scenario 1.

### 7.3 Scenario 3

**Fig 10:**
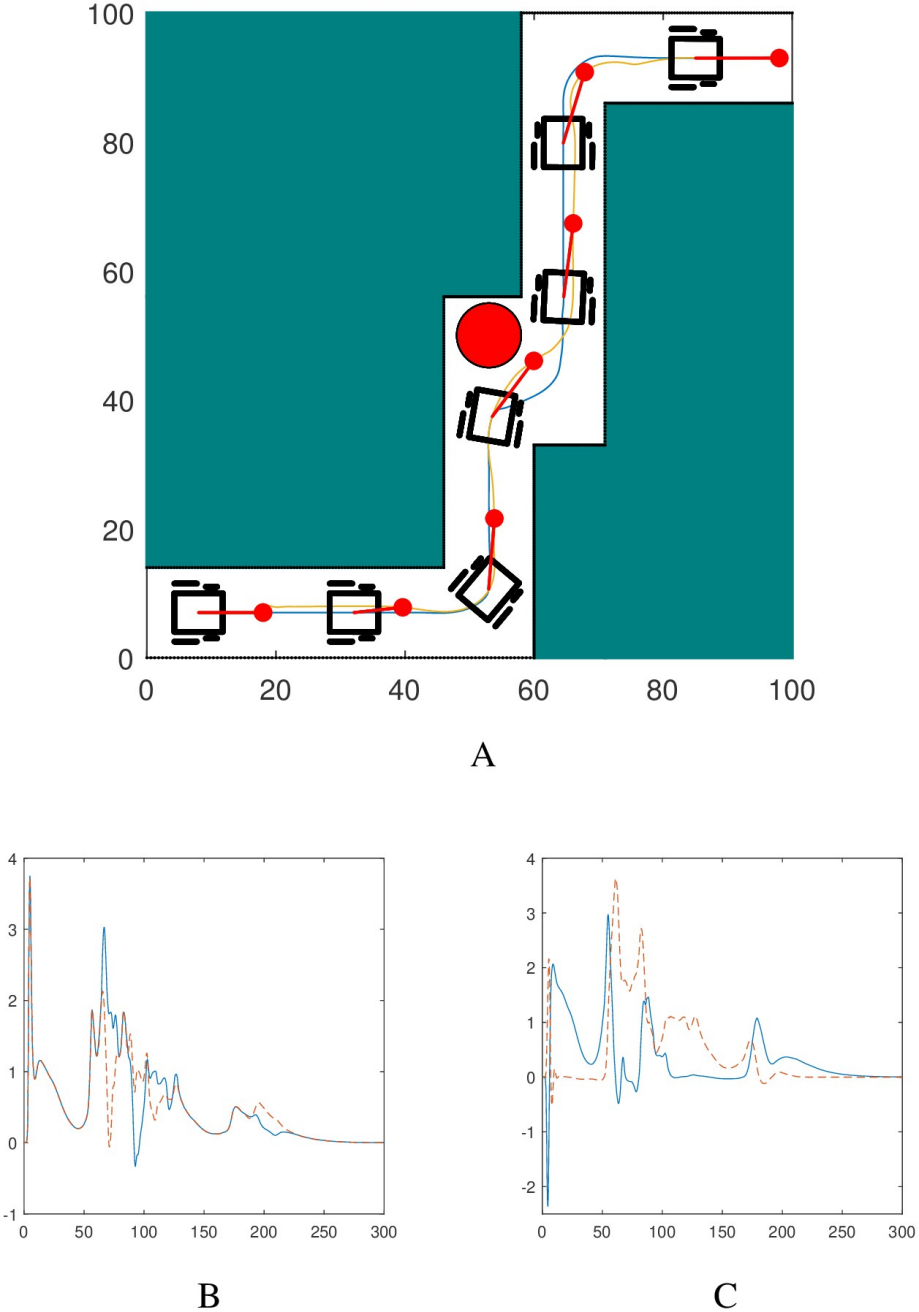
Evolution of trajectories of the robotic dog, wheelchair and the nonlinear velocities for the robotic dog wheelchair system along the trajectory shown in Scenario 3. A: Path followed by the robotic dog (in yellow) and the wheelchair robot (in blue). B: Velocities *v*_*R*_ in blue (solid), *v*_*L*_ in red (dashed). C: Velocities *u*_1_ in blue (solid), *u*_2_ in red (dashed).

Another intriguing scenario is simulated in Scenario 3 where the robotic dog wheelchair system travels between parallel line obstacles as shown in Fig 10A. The system avoids the side walls and the fixed circular obstacle along its path and finally converges at the target. This scenario emulates a real-life situation involving robots maneuvering inside a tunnel or pipe or even indoor paths such as narrow corridors. The evolution of the nonlinear velocities for the robotic dog wheelchair system are shown in Fig 10B and 10C. The velocities vanish as the system converges to the target. The behavior of the non-linear controllers and the Lyapunov function’s evolution and its time derivative are similar to that shown in scenario 1.

## 8 Discussion

Introduction of new and specialized assistive robots in health care continues to improve the physical and mental wellness of patients, the elderly, and individuals with disabilities (PWDs). Adaptation to these assistive robots ensures that patient-centered care will not be compromised, and there is a relief for shortage of competent nurses and a significant reduction in human errors. A collection of time-invariant, nonlinear, continuous acceleration control laws of an autonomous system, comprising of an assistive robot and a rehabilitation wheelchair robot, is presented in this paper while observing wheelchair’s restrictions and limitations. The control laws, which fall under the APF method, are derived from a total potential using LbCS. The control laws allow the assistive companion, robotic dog, guide the wheelchair robot during navigation in a highly constrained environment. The total potential function (or Lyapunov function in this case) designed from LbCS is built on the concept of potential gradient descent that ensures reaching to the bottom of the potentials. At every iteration while gradient of all possible paths are calculated, the path with the steepest descent is chosen. Therefore, the algorithm ensures reaching the bottom of the potentials through the shortest path. Also on a restricted domain, the Lyapunov function has continuous first partial derivatives which guarantee smoothness of the paths. The focus of this paper was just on the proof of concept, testing, demonstrating and validating effectiveness of the acceleration-based control laws. However, the future work will consider a quantitative analysis of the simulation results which can verify the effectiveness of the LbCS-based controllers. The simulation results, as depicted in Figs 5, 9 and 10, illustrate the efficacy and robustness of the controllers for collision-free navigation using a robotic dog as a virtual leader. However, the likelihood of algorithm singularities (local minima) is still present, a significant drawback of APF methods.

Moreover, as stated in [32], an assistive robot should ensure safety and rider comfort for rehabilitation. The acceleration control laws presented in this paper guarantee safety and user comfortability. The acceleration controllers will ensure that the user does not encounter abrupt shocks due to sharp velocities changes. However, the most recent assistive wheelchair robot presented in [21] will not provide a comfortable ride since it is velocity controlled; hence it will contain sharp changes in velocities.

The communication is assumed to be preprogrammed for daily routines by the supervisor in consultation with the people with disabilities riding the wheelchair. It is assumed that there is no input from the user during the journey. Thus, this research offers a distinctive solution to overcome the typical challenges faced by wheelchairs that require human inputs, particularly for individuals with disabilities who may be unable or incapable of directing the wheelchair. For instance, even the most basic mobility tasks, such as moving from one location to another, can be problematic for individuals with severe paralysis, mental impairments, comorbidities, musculoskeletal issues, and spinal cord injuries. In most cases another person has to be around as an assistive companion to control the motion of the wheelchair for the user for safe navigation. The new system proposed takes care of this situation by introducing a robotic dog on a virtual leash guiding the wheelchair to pre-determined destinations of users. To add on, for our autonomous system users do not require to provide any navigation commands since tasks to engage are controlled centrally and operationalized by the robotic dog while the wheelchair follows. Hence, the proposed system is well-suited for the user’s daily activities that can be programmed as central commands. For example, if the user typically takes an afternoon walk at a specific time, the system can be pre-programmed accordingly. However, for impromptu activities, the user or another individual (in cases where the user is unable) would need to interact with the robotic dog to issue navigation commands. Indeed there are various devices and tools in the literature such as the tongue device system, which can aid users in sending signals to the robotic dog.

The limitations of this study are:

- the kinematics and dynamics of a robotic dog’s links and limbs have yet to be modelled.
- the solution proposed in this study is only feasible in a highly constrained and isolated human environment where nothing else appears to be moving in the direction of the heterogeneous system.
- the presence of algorithm singularities in the form of local minima as LbCS is based on the classical method of the artificial potential field approach. In this study, such cases where the system could get trapped in a local minima were avoided through the selection of specific initial conditions and assigning specific values to the control, convergence and avoidance parameters using brute force technique.
- the authors have restricted themselves to using numerical proofs and computer-based simulations of interesting scenarios to demonstrate the effectiveness of the acceleration-based control laws. This paper provides a theoretical exposition of the LbCS’s applicability only and the acceleration control laws achieved were not integrated onto some prototype experimental robot for practical results.
- the control, convergence and avoidance parameters used have not been optimized.
- the control laws achieved cannot address a dynamic environment.
- correct multi-variable analysis, which could help in developing a more accurate model by understanding the relationships between multiple variables used.

## 9 Conclusion

A set of two-dimensional stabilizing nonlinear time-invariant continuous control laws were proposed for a newly designed autonomous robotic dog and rehabilitation wheelchair system. An assistive robot seen as a robotic dog guides the wheelchair robot in a highly constrained and isolated human environment where nothing else appears to be moving in the direction of the heterogeneous system to the pre-determined target of the user. The application of the nonlinear control laws to the wheelchair robot, which is subject to its kinematic equations, allows it to follow the robotic dog towards a desired location, while adhering to the constraints and limitations of the system. The robotic dog successfully guides the wheelchair robot during the navigation process in a cluttered environment where the avoidances are from the robotic dog and the integrated dynamic protective polygon. The wheelchair with its own path traverses the obstacle-free region traced by the dynamic polygon. The leash is flexible, and its length is bounded, which invariably provides the protective polygon to change its intrinsic dimension. Hence, the heterogeneous system has increased mobility for obstacle avoidance and passing through narrow passageways. Finally, the acceleration based controllers guarantee the user’s comfort by avoiding sudden changes in the wheelchair’s wheel angular velocities. According to the author, this is the first time such stabilization control laws have been developed for an autonomous robotic dog and rehabilitation wheelchair system.

This paper focuses on the theoretical feasibility of LbCS and aims to demonstrate the effectiveness of control laws through computer simulations and numerical proofs in various scenarios. However, a drawback of this approach is the possibility of introducing algorithm singularities or local minima. In real-world applications, continuity must be discretized, and only asymptotic stability can be ensured. Nevertheless, incorporating such controllers is practical for the industry to create autonomous systems in the healthcare sector, which may comprise assistive robots for personal rehabilitation and robots for transportation. For future works, a hybridization of the control scheme using one of the heuristic -based approaches such as Ant colony optimization will be looked into to remove the problem of local minima and combining the strengthens of the two approaches to address other related issues such as moving obstacles. Furthermore, as for future research it is also essential to statistically compare the experimental results achieved using the approach used with other existing techniques in the literature on key performance indicators like path length and convergence time.

## Supporting information

S1 File (RAR)
